# Efficient Conversion of Glucose to Methyl Lactate with Sn-USY: Retro-aldol Activity Promotion by Controlled Ion Exchange

**DOI:** 10.1021/acssuschemeng.2c01987

**Published:** 2022-06-28

**Authors:** Jose M. Jimenez-Martin, Ana Orozco-Saumell, Héctor Hernando, María Linares, Rafael Mariscal, Manuel López Granados, Alicia García, Jose Iglesias

**Affiliations:** † Chemical & Environmental Engineering Group, Universidad Rey Juan Carlos, C/ Tulipan s/n, 28933 Madrid, Spain; ‡ Energy and Sustainable Chemistry (EQS) Group, Institute of Catalysis and Petrochemistry, CSIC, C/ Marie Curie 2, Campus de Cantoblanco, 28049 Madrid, Spain; § IMDEA Energy Institute, Av. Ramón de la Sagra 3, 28935 Móstoles, Madrid, Spain

**Keywords:** tin, USY, Sn-USY, zeolite, ion exchange, retro-aldol condensation, alkyl lactate, lactic acid, glucose

## Abstract

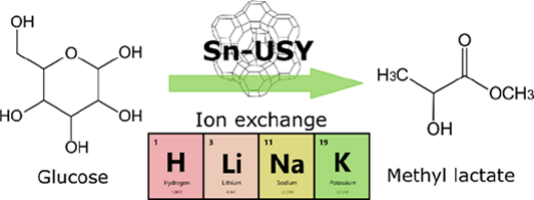

Sn-USY materials have been prepared through an optimized post-synthetic catalytic metalation procedure. These zeolites displayed, upon ion exchange with alkaline metals, an outstanding activity in the direct transformation of glucose into methyl lactate, yielding more than 70% of the starting glucose as the target product, and an overall combined retro-aldol condensation product yield above 95% in a short reaction time (<4 h). This outstanding catalytic performance is ascribed to the neutralization of Brønsted acid sites, the consequent depression of side reactions, and a higher population of tin open sites in the ion-exchanged Sn-USY zeolites. Reusability tests evidenced some loss of catalytic activity, partially caused by the closing of tin sites, although the use of small amounts of water in the reaction media demonstrated that this deactivation mechanism can be, at least, partially alleviated.

## Abstract 2

K-exchanged Sn-USY zeolites prepared by post-synthetic metalation provide extraordinary catalytic performance in the one-pot transformation of glucose to methyl lactate, showing product yields above 70%.

## Introduction

Tin-containing zeolites display high catalytic activity in a multitude of Lewis acid driven transformations involving a wide collection of substrates.^1−6^ This extraordinary catalytic performance in a large variety of chemical transformations^7^ has allowed applying these zeolites in the promotion of reaction cascades to produce γ-valerolactone^8^ or lactic acid/alkyl lactates. This last transformation has received much attention in recent years^9^ mainly because of the interest on developing sustainable, efficient, chemocatalytic routes to lactic acid, which would eventually overcome the troubles associated to its industrial production through fermentation.^10^ The development of Sn-β zeolite^11^ and its application to the one-pot transformation of sugar monosaccharides into alkyl lactates^12^ are the two major breakthroughs in this area. However, several studies suggest that other Sn-containing zeolites, such as MWW^13,14^ or FAU-type^15−17^ zeolites, also display high catalytic activity in several transformations.^17^ Indeed, some zeolite structures are easier to prepare at a high scale than β materials, making them more accessible and attractive options as starting materials for the preparation of tin-containing zeolite catalysts.

The synthesis of some types of tin-containing zeolites can be achieved by hydrothermal crystallization, yielding defect-free zeolites.^11,18^ However, these procedures involve difficult and long hydrothermal treatments, resulting in limited metal loadings and the formation of large zeolite particles, which can cause diffusional limitations.^18^ Post-synthetic zeolite metalation procedures typically consist of two steps in which the dealumination and/or desilication of the starting zeolite creates vacancies within the crystal framework, which are occupied by metal species in a second (metalation) step.^13,19,20^ Post-synthetic preparation methods not only are simpler than hydrothermal crystallization procedures when considering tin-containing zeolites (these can be completed in only few hours) but also allow the incorporation of higher metal loadings and avoid the use of expensive organic templates.^21,22^ Nonetheless, tin species display a different speciation as a function of the preparation method.^23^ Tri-coordinated tin sites ((≡SiO)_3_Sn-OH), usually described as open tin sites, are plentiful in materials prepared by post-synthesis methodologies, whereas tetrahedrally coordinated tin atoms, known as closed sites ((≡SiO)_4_Sn), are more abundant in hydrothermally crystallized materials.^24^

Numerous studies have focused on ascertaining the nature of open and closed Sn sites in tin zeolites and on establishing correlations between the properties of these metal sites and their intrinsic catalytic activity.^25−29^ Experimental^28,30^ and quantum chemical calculations^31^ have demonstrated that open sites are more flexible and stronger Lewis acid centers than closed sites, thus resulting in more active catalysts. However, both types of tin centers can be interconverted by the simple addition of water molecules to/from the vicinity of metal species.^26^ This modification is accompanied by the generation of strong Brønsted acidity by the interaction of water with open tin sites,^32^ which promotes side transformations. To prevent such a negative effect, alkaline species have been used as additives in the reaction media to neutralize Brønsted acids in Sn-zeolites,^33−35^ increasing their catalytic performance toward the selective formation of alkyl lactates. Our previous work evidenced that this step can be conducted prior to the reaction by ion exchange,^36^ although with a limited performance because of the poor control of the Sn and alkali cation loading finally supported within the Sn-zeolite catalyst. Within this work, we have conducted a thorough study on the incorporation of both tin and alkali cations to USY zeolites, looking for a careful control of the incorporation of both types of species. The more advanced synthesis of the Sn-USY material has been improved by the application of a chemically assisted grafting procedure. On the other hand, using dilute solutions of several alkali chlorides (0.5 mol·L^–1^; Li, Na, and K) and multiple ion exchange steps allows for an efficient control of the final loading of alkali cations and the tuning of the Brønsted/Lewis acidity of the final materials. This allows producing Sn-USY materials with an outstanding catalytic activity in the one-pot conversion of quite concentrated methanolic solutions of glucose (ca. 5.7 wt %), yielding 70% of the starting sugar as methyl lactate, an extraordinary catalytic performance not previously described.

## Results and Discussion

### Synthesis of Sn-USY Zeolites

Table S1 lists the main physicochemical properties recorded for the prepared zeolite samples, including metal contents and textural and acid properties. The synthesis of Sn-USY materials starts with the dealumination of the parent zeolite. The overall high efficiency of dealumination is supported by the low Al content found in the zeolite after the acid treatment. HNO_3_ removes 90% of the starting Al content present in the parent material, but it requires a second cycle to achieve low aluminum contents. ^27^Al NMR spectra (Figure S1) show that the parent USY zeolite contains both intra- (FAL) and extraframework (EFAL) aluminum species, adopting tetrahedral and octahedral coordination, respectively. The first dealumination step with HNO_3_ removes a substantial fraction of the starting Al content, with the removal of extraframework aluminum species being more intense due to their lability. The second HNO_3_ treatment allows achieving a complete removal of the EFAL and the vast majority of the intraframework aluminum atoms. Nevertheless, a fraction of the starting Al content (ca. 0.6 wt %) remains attached to the zeolite framework, as evident from the signal detected at 59.6 ppm in ^27^Al NMR spectra. These species, due to the broadening of the signal and shift toward lower chemical shift values, might be ascribed to intraframework tetrahedral Al sites being highly distorted by the interaction with charged EFAL atoms, so a low Brønsted acid capacity is expected in these materials.

Regarding the incorporation of tin species, metalation provided a high tin loading involving an almost quantitative incorporation of Sn for all the tested metal loadings (1, 2, and 4 wt %). These results point to a higher efficiency of the TEA catalyzed metalation process^37^ as compared to other routes based on thermal treatments.^24^ The structural properties of the prepared materials have been assessed by XRD analysis. Recorded diffraction patterns (Figure S2A) correspond to the faujasite crystal structure, with all the diffractions well defined and reproduced in the tested materials. The crystalline structure was not significantly affected during the dealumination and metalation steps. Nevertheless, a slight shift of the diffractions toward higher angle values was observed after dealumination, indicating the decrease of the unit cell size of the zeolite because of the removal of intraframework Al sites. Metalation produced the opposite effect because of the incorporation of Sn, a much larger metal atom than silicon or aluminum. However, the initial cell size of the USY parent material was not fully recovered because the incorporated tin loading was much lower than the amount of extracted aluminum. No other diffractions were observed in the collected patterns despite the high tin loading, so the presence of large crystalline domains of SnO_2_ can be discarded.

The textural properties of zeolites have been evaluated by gas adsorption–desorption analysis (Table S1). Ar adsorption–desorption isotherms recorded for the parent and Sn-metalated USY zeolite (Figure S2B) were identified as type I isotherms according to the IUPAC classification, which are typical for microporous materials. The isotherms featured H3 hysteresis loops, which can be attributed to the adsorption of argon onto the interparticular voids of the tested samples. The differences between the textural properties recorded for the parent and Sn-functionalized USY zeolites are quite small. Sn-USY materials displayed a slight increase of the BET surface area and total pore volume, probably as a consequence of the formation of additional mesoporosity occurring during the dealumination step,^15^ but pore size distributions remained almost unaltered in the micropore region (Figure S2C). In this way, the damage of the porous structure of the zeolite can be discarded during the dealumination and metalation steps.

**Figure 1:**
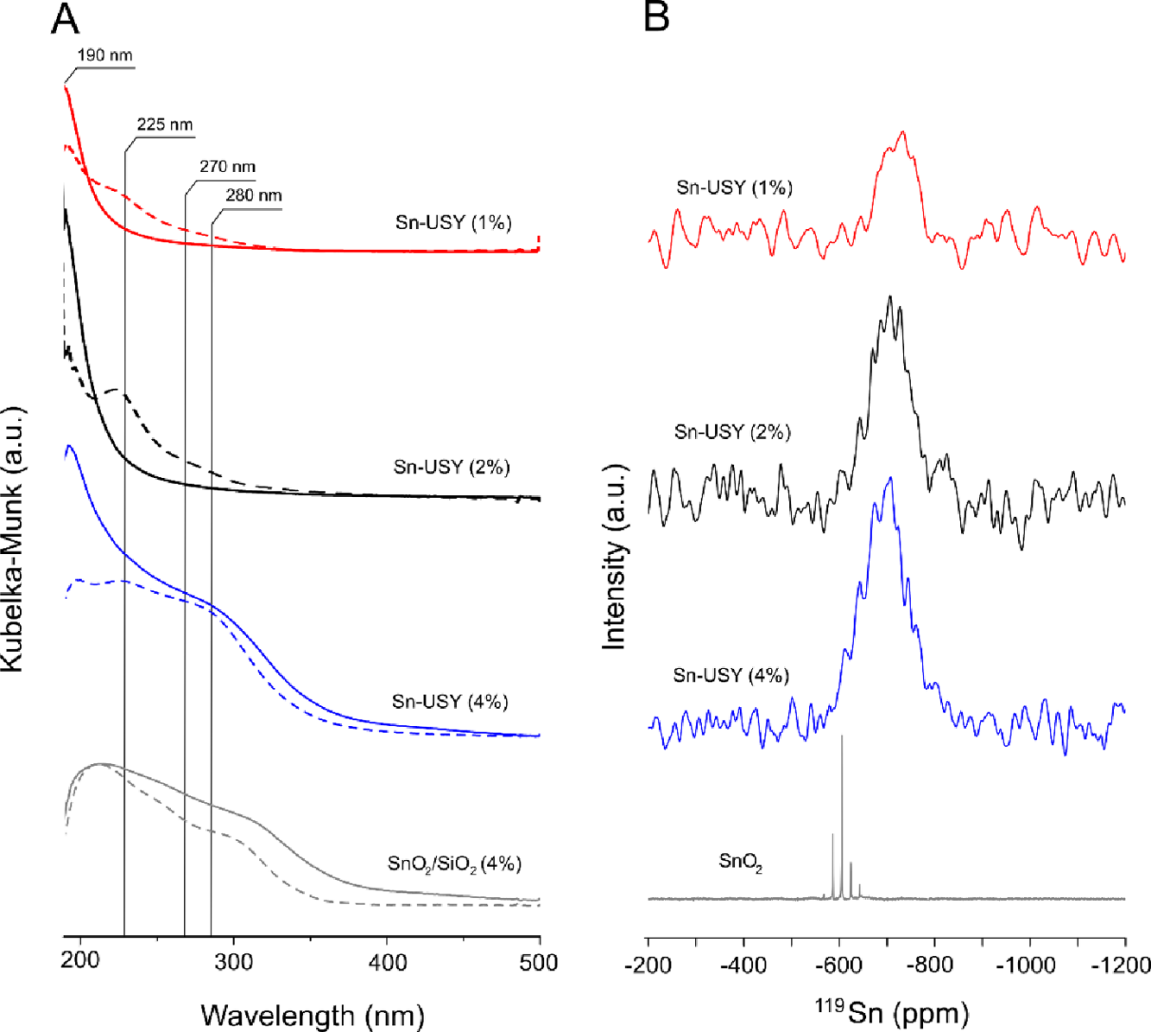
(A) DR UV–vis spectra recorded for samples prepared with different metal loadings before (dash lines) and after (solid lines) thermal treatment at 350 °C for water removal. (B) ^119^Sn solid-state MAS NMR results for the same samples.

**Figure 2:**
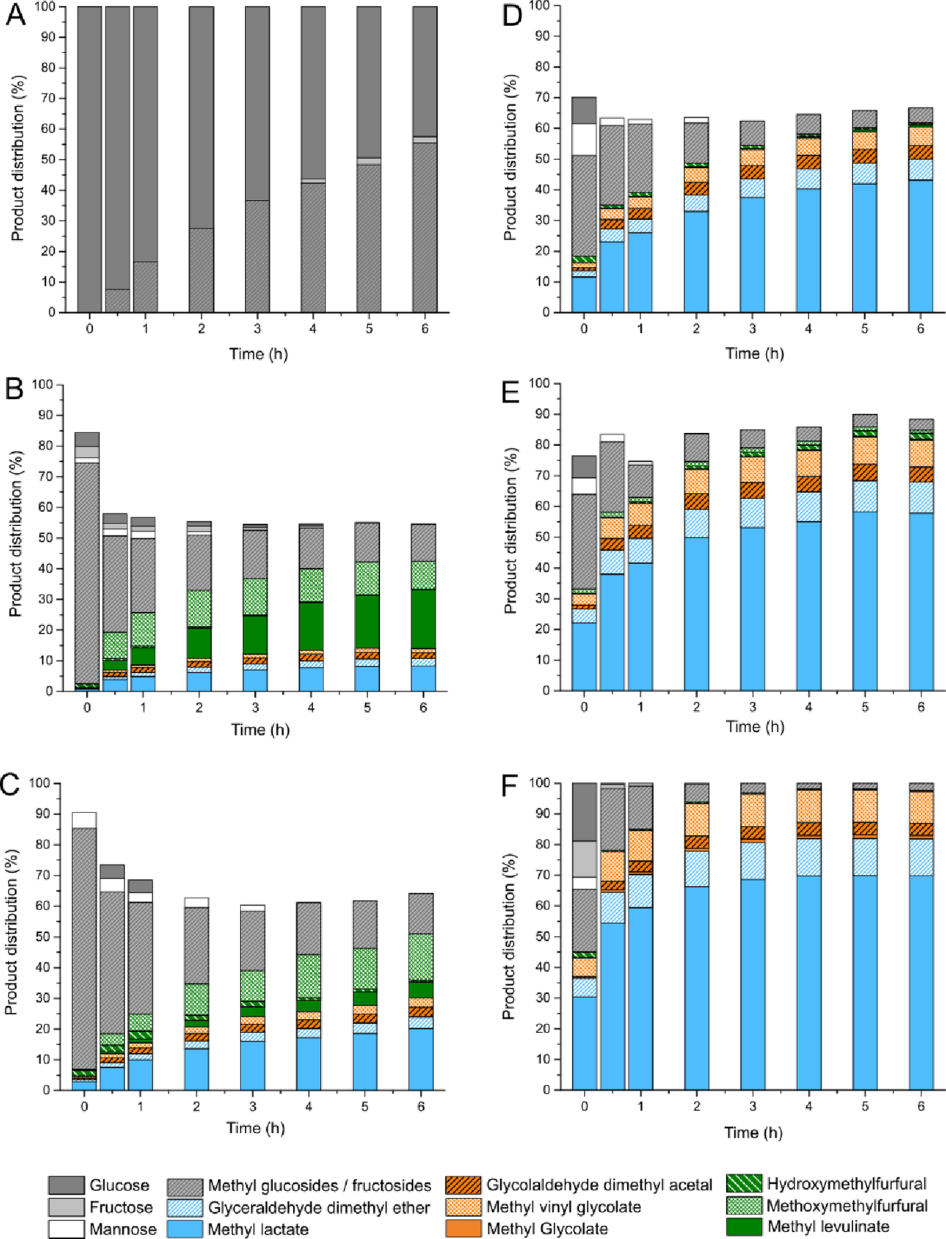
Results obtained from catalytic tests performed on methanolic solutions of glucose in the presence of the (A) blank reaction, (B) as-made Sn-USY, (C) hydrated Sn-USY, (D) [Li]Sn-USY, (E) [Na]Sn-USY, and (F) [K]Sn-USY. Reaction conditions: reaction volume, 75 mL; catalyst loading, 0.75 g; substrate concentration, 266 mM; and reaction temperature, 150 °C.

The environment of the zeolite-grafted tin species has been assessed by means of DR UV–vis spectroscopy. Figure 1A depicts the DR UV–vis spectra recorded for the synthesized Sn-USY materials before and after *in situ* dehydration at 350 °C. UV spectra recorded for the untreated samples (Figure 1A, dash lines) are dominated by the presence of three distinct UV absorption bands located at ca. 190, 225, and 270–300 nm. The signal located at 190 nm is attributed to the ligand-to-metal charge transfer (LMCT) from oxygen atoms to Sn^4+^ sites in tetrahedral coordination within the matrix support. The high energy associated to this charge transfer suggests that tin sites occupy locations at the zeolite matrix with low electron-donating capacity, and thus, a higher positive charge and a subsequent stronger Lewis acidity are expected on these tin sites.^28^ On the other hand, lower-energy signals, such as those detected at 225 and 270–300 nm, are usually assigned to the presence of tin sites in higher coordination numbers. This could be attributed either to the presence of tin dioxide domains or to the adsorption of polar (water) molecules and their coordination to tin sites. However, the absence of SnO_2_ signals in XRD patterns points to a large dispersion of the tin species and to SnO_2_ domains that, if present, are small enough not to provide reflections in XRD. The *in situ* dehydration of Sn-USY samples in the UV spectrometer prior to their analysis (Figure 2A, solid lines) led to the removal of the low-energy absorption bands for samples prepared with 1 and 2 wt % Sn contents. This result indicates that the origin of these UV bands is most likely due to the adsorption of water molecules onto tin sites, leading to hydrated forms of isolated Sn^4+^ species.^29,38^ On the contrary, the sample showing the highest metal loading (4 wt % Sn) displays a completely different behavior, as the absorption located at 270–300 nm was still present after *in situ* dehydration treatment. This result evidences the presence of tin species adopting a permanent octahedral coordination, such as tin oxide.

To get a better insight into the local environment of tin atom sites, ^119^Sn SS MAS NMR experiments were performed on the Sn-functionalized zeolites (Figure 1B). Spectra reveal the presence of a signal located in the region from −705 to −720 ppm, conventionally attributed to hydrated tin sites in tetrahedral coordination, in all the prepared samples.^26^ The absence of defined signals around −605 ppm, conventionally ascribed to bulk tin dioxide, indicates that there is no presence of large domains of tin oxide in the prepared materials,^26^ at least for samples prepared with low metal loadings. However, resonance signals in the ^119^Sn SS MAS NMR widened and shifted toward lower chemical shift values, suggesting that tin sites display a higher average coordination number as the tin loading increases. In this sense, the sample containing the highest tin loading (4 wt %) is wide enough to cover chemical shifts ascribed to tin dioxide domains, as previously detected through DR UV–vis spectroscopy. This result is in agreement with the results provided by Hermans et al.^39^ who demonstrated that large tin loadings supported onto dealuminated β zeolite lead to the formation of tin dioxide, though in a quite small fraction. In this way, the combination of DR UV–vis spectroscopy and ^119^Sn SS MAS NMR evidences the incapability of dealuminated USY zeolite to accommodate such a high metal loading as 4 wt % in the form of isolated tin sites. Thus, the Sn-USY zeolite with 2 wt % Sn loading was the sample showing a highest metal incorporation as isolated tin species, so the rest of this work was performed using this material.

The synthesis of the catalysts was completed by ion exchange of the Sn-USY materials with different alkaline metal chlorides. For this purpose, Sn-USY prepared with 2 wt % Sn was used as the starting material. Ion exchange Sn-zeolites with alkali cations have been described to passivate Brønsted acid sites,^33^ thus revealing the catalytic activity of Sn sites in retro-aldol condensation routes.^36^ We postulate that the activation of Sn sites occurs by the partial hydrolysis of the metal centers resulting in open tin sites followed by ion exchange of the alkali cation in the created Brønsted acid site caused by the interaction of water with tin sites. In this way, the water-induced Brønsted acidity is avoided,^32^ and the closing of the tin site is prevented by the interaction of the alkali cation with the Si-OH groups created in the vicinity of tin sites.^5^ Metal contents, monitored by ICP-OES, confirm the presence of all the tested alkali cations into the Sn-zeolite after ion exchange. Nevertheless, the amount of alkali metal loading increased with the size of the cation, so a larger atom ratio of alkali metal to Sn was found for K as compared to Na and Li. This result points to a better performance of K in the removal of Brønsted acidity in Sn-USY, and thus, a better catalytic performance is expected for this material.

### Catalytic Performance

Tin-containing USY zeolites have been tested as catalysts for the production of methyl lactate from glucose using methanol as the solvent. Figure 2 depicts the product distributions achieved in several catalytic tests performed in the presence of Sn-USY zeolites. This study comprises a blank reaction (Figure 2A); two tests conducted in the presence of the parent Sn-USY zeolite (2 wt % Sn): after calcination (Figure 2B) and after hydration in water (Figure 2C); and the catalytic assays performed in the presence alkali cation-exchanged samples prepared with lithium, sodium, and potassium chlorides (Figure 2D–F). Detected products correspond to reaction routes described in Scheme S2.

The blank test led to a very high substrate conversion, where 46% of the starting glucose is transformed after reacting for 6 h in methanol at 150 °C. In this experiment, a mixture of methyl glycosides (methyl glucoside, methyl fructoside, and methyl mannoside), which are the result from the condensation of methanol with monosaccharides, is detected. These transformations are catalyzed by the presence of mild acids,^40^ but it is evident that, under the tested reaction conditions, the extension of glycosidations is massive, so these products are expected to occur in all the catalytic tests. No evidence of other products is detected, confirming that either glucose hydrolytic pathways, providing 5-hydroxymethyl furfural (5-HMF) and methyl levulinate, or retro-aldol condensation transformations, to provide methyl lactate, do not occur in the absence of the catalyst. Calcined Sn-USY also produced methyl glycosides as main products, which are obtained at the maximum yield (ca. 73%) during the early stages of the reaction. This result reflects quite a high catalytic activity of the tin-containing USY zeolite in glycosidation reactions, similarly to Sn-β zeolites,^41^ as a consequence of the Lewis acidity provided by Sn sites. Together with methyl glycosides, fructose and mannose coming from the isomerization and epimerization of the starting glucose were also detected but in low amounts. As for the evolution of the reaction media, Sn-USY zeolite led to the rapid consumption of the evolving methyl glycosides, together with the formation of methoxy methyl furfural (MMF) and methyl levulinate in quite appreciable yields at 6 h (9.2 and 19.1% molar yields, respectively). On the contrary, products coming from retro-aldol condensation routes were detected in a minor concentration, which include glycoladehyde dimethyl acetal (GADMA, 1.9% yield) and methyl lactate (8.3%) as main representatives of this pathway. These results reveal the occurrence of a complex reaction network in which glucose can undergo several reaction pathways, including glycosidation, isomerization, dehydration, and retro-aldol condensation transformations, among others (Scheme S2). Nevertheless, these results also evidence the poor catalytic performance of the Sn-USY material, which displayed negligible activity in retro-aldol sugar conversion pathways and a preference to promote hydrolytic transformations. These transformations are accompanied by some other side reactions resulting in unknown products, such as humins, produced through Brønsted acid driven pathways. In this way, Sn-USY material provides poor carbon balances and a huge amount of unknown products.

Hydrating the Sn-USY zeolite, using a similar treatment to that described for ion exchange (50 °C, 2 h; air-calcined), led to the partial opening of tin sites, exerting a notable influence on the catalytic efficiency of this material. This sample provided a rapid substrate conversion together with a different product distribution as compared to the as-made Sn-USY material. Higher yields toward retro-aldol condensation derived products were achieved, yielding 20.2% of the starting glucose as methyl lactate. On the contrary, hydrolytic route derived products were detected in a lower concentration, with methoxy methyl furfural (MMF) being the main product obtained in this pathway. These results suggest that the hydration of Sn-zeolites, which causes the opening of the tin sites, leads to the activation of retro-aldol condensation routes while depressing Brønsted acid driven reaction pathways, such as sugar dehydration or hydrolytic transformations. In this way, the amount of unknown products was partially reduced as compared to the parent Sn-USY material, although they were still extensively produced.

Conducting the same treatment as in hydrated Sn-USY but in the presence of alkali chloride salts (ion exchange treatments) enhanced the modification of the catalytic efficiency described for the hydrated catalysts. All the samples treated with alkali cations exhibited a negligible production of hydrolytic route derived products, like HMF, MMF, or methyl levulinate (Scheme S2). This result suggests that, as expected, Brønsted acidity in Sn-USY has been passivated during the ion exchange treatment, suppressing its activity in the promotion of hydrolytic routes. On the contrary, very high yields of retro-aldol condensation derived products were obtained, with methyl lactate being the major product in all the cases (43% for [Li]Sn-USY, 57.8% for [Na]Sn-USY, and 70.0% for [K]Sn-USY, all of them at 6 h). This outstanding catalytic performance has not been previously reported, and this work demonstrates the feasibility to efficiently conduct the transformation of glucose into methyl lactate using Sn-faujasite-type zeolites because of their extraordinary catalytic performance in sugar retro-aldol condensation. Indeed, together with methyl lactate, ion-exchanged Sn-USY zeolites provided some other products coming from retro-aldol condensation pathways (Scheme S2). Methyl glycolate (MG), glycolaldehyde dimethyl acetal (GADMA), or methyl vinyl glycolate (MVG) is produced through the retro-aldol condensation of glucose, which is split into a C2 and a C4 carbon backbone moieties. Finally, besides glucose derived products, glyceraldehyde dimethyl ether (GDME) is also detected. In this case, we postulate that this C3 carbon backbone compound is produced through the retro-aldol condensation of methyl fructoside, one of the methyl glycosides evolving during the beginning of the reaction. The sum of the product yields achieved through retro-aldol condensation routes in the presence of [K]Sn-USY ascends to 97.4% of the starting glucose loading, which is a strong evidence of the very high catalytic efficiency of this potassium exchange zeolite in the promotion of carbohydrate retro-aldol condensation. Differences between lithium-, sodium-, and potassium-exchanged Sn-zeolites are reflected in the activity of the catalysts, specifically in the production of methyl lactate. The lithium-exchanged zeolite provided quite a high lactate yield (43.1%) but with poor selectivity, as evident from the large amount of unknown products formed in the reaction. Selectivity to methyl lactate was much higher in the case of the sodium-exchanged zeolite and in the potassium-treated material, for which the sum of all the detected products allowed closing the mass carbon balance. These differences correlate with the acidity of the different zeolites evaluated by means of NH_3_-TPD analysis, with the results being summarized in Table S1.

The acidity of the parent H-USY material is very high (3.39 mEq H^+^·g^–1^) as a consequence of the protonic form in which this zeolite is used. The interexchange of aluminum by tin species during dealumination and metalation stages led to a substantial decrease in acid capacity (2.78 mEq H^+^·g^–1^) and strength, as evidenced by the lower ammonia desorption temperature detected for sample Sn-USY (280 °C) as compared to H-USY (318 °C) (Figure S3). This difference is attributed to the removal of Brønsted acidity during dealumination, which is not compensated by the incorporation of tin sites. Ion exchange with lithium, sodium, and potassium cations resulted in less acidic materials, demonstrating that a fraction of the acid sites present in the parent Sn-USY zeolite is passivated in this stage. Acidity reduction of Sn-USY by ion exchange is seemingly linked to the size of the alkali metal cation (the larger the ion radius is, the larger is the basicity), so acidity reduction followed the order K > Na > Li. The final acidity of the materials correlates with the catalytic results provided by the Sn-USY zeolites, so depressing the acidity results in a lower extension of side reactions, a better carbon balance, and a higher selectivity for methyl lactate.

With the aim of enhancing the activity of Sn-USY materials in the promotion of glucose transformation to methyl lactate, cation exchange with potassium chloride was intensified. For this purpose, up to four different consecutive ion exchange cycles were completed, repeating the same procedure already described over the Sn-USY material. Table S1 lists the metal composition present in Sn-USY materials treated with different ion exchange cycles with KCl (Table S1, entries 5–8). Both aluminum and tin contents remained unaltered in the course of the different cycles, meaning that these species were highly stable and did not easily leach during the ion exchange treatments. The successive ion exchange cycles led to increasing potassium loadings, thus raising the K/Sn atom ratio from 0.64 for sample [K]Sn-USY to 1.23 for [Kx4]Sn-USY. The consequences of the incorporation of potassium onto the properties of Sn-USY materials were evaluated by means of different techniques. Nitrogen adsorption revealed that the porous structure of the material was well preserved (Table S1). On the contrary, the acid/basic properties of the materials were modified. Figure S4 depicts the results achieved from NH_3_ and CO_2_ thermal programmed desorption experiments conducted on Sn-USY and the K-exchanged materials. Ammonia TPD profiles (Figure S4) reflect a decrease in acid capacity from 2.78 mEq H^+^·g^–1^ recorded for Sn-USY to 1.75 mEq H^+^·g^–1^ detected in [K]Sn-USY and to 1.08 mEq H^+^·g^–1^ in sample [Kx4]Sn-USY. As for the strength of the remaining acid sites, only the first exchange caused a decrease in the NH_3_ desorption temperature from 280 °C for Sn-USY to around 260 °C for [K]Sn-USY. Consecutive ion exchanges with potassium did not affect the desorption temperature of ammonia, remaining constant regardless of the number of cation exchange cycles. We presume the first ion exchange passivates strong Brønsted acid sites, which could be attributed to the remaining intraframework aluminum sites with ion exchange capacity. The rest of the ion exchange cycles incorporate potassium to the Sn-USY materials, probably in passivating the Brønsted acid sites created during the ion exchange by interaction of Sn sites with water.^42^

Incorporation of potassium in USY zeolites is well known to provide basicity, so CO_2_ TPD has been used to evaluate the basic properties of the K-exchanged zeolites (Figure S4). Despite the potassium loading reached in zeolites, the basic capacity decreases with each ion exchange. These results provide evidence that the incorporation of potassium might be occurring on different sites depending on the ion exchange cycle. The large reduction of acidity in Sn-USY when ion-exchanged for the first time and the associated increase in basicity suggest that Brønsted acid sites, such as those linked to the remaining aluminum sites in USY zeolite, are passivated in the first term. However, as indicated by the ^27^Al SS MAS NMR results, most of the aluminum presents a distorted environment liable to be caused by intraframework Al sites interacting with EFAL species, so its ion exchange capacity is considered very low. Further incorporation of potassium seems to proceed in a different way, probably by interaction with tin sites by passivation of the aforementioned Brønsted acid sites created by the interaction of Sn with water molecules.^42^ To evaluate this possibility, K-exchanged zeolites have been characterized by means of infrared spectroscopy.

FTIR spectra recorded for Sn-USY zeolites in the OH stretching region are shown in Figure S5. The spectrum recorded for the Sn-USY parent material depicts several contributions attributed to the presence of hydroxyl functionalities: isolated external and internal silanols (3740 and 3730 cm^–1^, respectively), extraframework Al-OH (3690 cm^–1^), Si-O(H)-Al Brønsted acid hydroxyls in the faujasite structure (3630 and 3560 cm^–1^),^43^ and hydrogen-bonded internal silanols (3500 cm^–1^). The ion exchange treatment of the Sn-USY with potassium chloride leads to, in the first term, the removal of the acidic Brønsted hydroxyls, indicating that these species are the first to be passivated. The successive ion exchange cycles resulted in the removal of both isolated and H-bonded internal silanols, which could be caused by either the thermal condensation of the hydroxyl functionalities leading to siloxane bridges or the ion exchange of the more acidic silanols by potassium.

**Figure 3:**
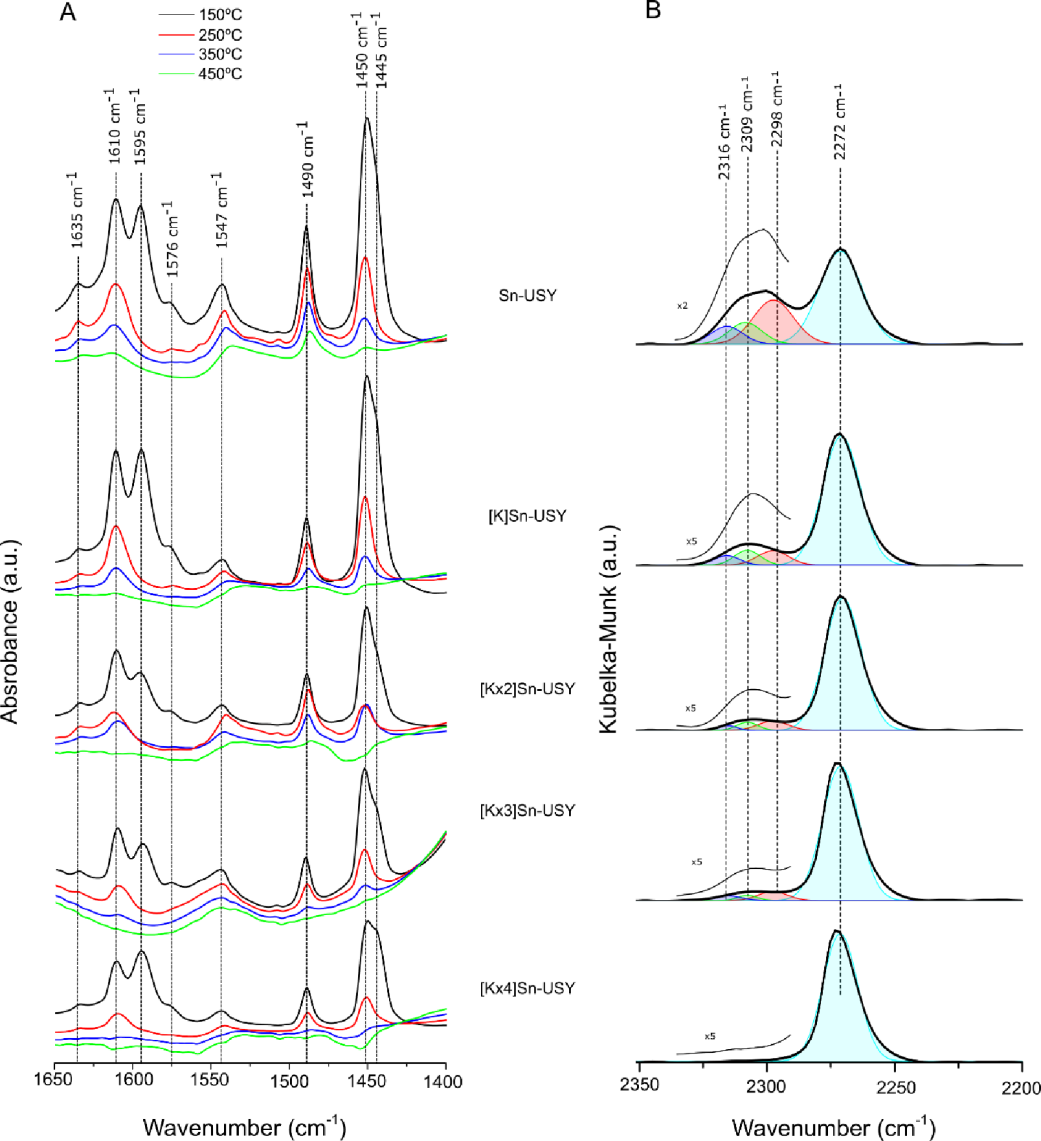
(A) FTIR spectra of pyridine and (B) DRIFT spectra of deuterated acetonitrile adsorbed on the Sn-USY material and samples prepared by K-exchange with different cycles.

The acidic properties of the Sn-USY material and the K-exchanged samples prepared thereof have been evaluated by means of FTIR spectroscopy combined with the use of molecular probes (pyridine and deuterated acetonitrile), with the results depicted in Figure 3. Pyridine adsorption FTIR spectra recorded after adsorbing pyridine onto the Sn-USY zeolites (Figure 3A) have been recorded after evacuation at different temperatures (150–450 °C) to qualitatively ascertain the acid strength of the acid species. After the adsorption of pyridine and evacuation at 150 °C, all the samples showed characteristic vibration bands ascribed to the adsorption of pyridine onto different types of Brønsted and Lewis acid sites. Signals at 1635 and 1545 cm^–1^ are usually attributed to H-bonded pyridine in strong Brønsted acid sites (BAS), whereas signals located at 1610 and 1452 cm^–1^ correspond to pyridine adsorbed onto strong Lewis acid sites (LAS), e.g., coordinatively unsaturated Al^3+^ species. Weak acid sites, like those located at 1595 and 1445 cm^–1^ (H-bonded Py) and 1575 cm^–1^ (weak Lewis site coordinated Py), are also detected.^44^ Finally, the signal detected at 1490 cm^–1^ is ascribed to the adsorption of pyridine on both weak Lewis and Brønsted acid sites.

Regarding the acid capacity ascribed to both Brønsted and Lewis sites, Table S2 lists the concentration of each species detected at the tested evacuation temperatures, which was calculated by direct titration with pyridine as reported by Emeis.^45^ Sn-USY shows the highest population of strong BAS, which is also evident from the higher intensity of the signals detected at 1545 cm^–1^ for this material, regardless of the evacuation temperature. The different ion exchange cycles with KCl progressively reduces the intensity of this signal, indicating that K-exchange passivates these acid species. Regarding Lewis acidity, the consecutive incorporation of potassium to the Sn-zeolites leads to higher BAS to LAS ratios. This result suggests that not only Brønsted acid sites are affected by the incorporation of K cations but also Lewis acid sites. A detailed evaluation of the Lewis acidity by comparing the normalized concentration of LAS sites^46^ vs the ion exchange cycle and vs the evacuation temperature (Figure S6) evidences the negative influence of the potassium incorporation on the relative Lewis acid capacity. The first K-exchange treatment does not affect much the Lewis acidity of the Sn-USY zeolite, but after the second ion exchange cycle, the Lewis acidity drops to half of its initial capacity (Figure S4A). Moreover, ion exchange with potassium not only reduces the concentration of Lewis acid sites but also reduces their strength, as can be ascertained when comparing the relative Lewis acid capacity vs the evacuation temperature during the recording of FTIR analyses after pyridine adsorption (Figure S6B). These results might be pointing to an eventual interaction taking place between the potassium species incorporated by ion exchange treatments and the previously grafted tin sites. Thus, the incorporation of potassium not only passivates Brønsted acidity, but it also could compromise the Lewis acidity of the Sn-USY materials. To explore this possibility, DRIFT spectra of adsorbed deuterated acetonitrile on Sn-USY and potassium-exchanged zeolites were used to characterize the Lewis acidity provided by the supported tin species and their evolution with the potassium incorporation by ion exchange.

Figure 3B depicts the DRIFT spectra recorded for the Sn-USY zeolites. Different signals are detected in the region 2400–2200 cm^–1^, which are attributed to the stretching of the nitrile group of deuterated acetonitrile adsorbed onto different acid sites. The main vibration, located at 2272 cm^–1^ and present in all the tested materials, is conventionally attributed to CD_3_CN molecules adsorbed onto weak BAS like silanol groups.^47^ These are plentiful in all the samples, as these have been prepared involving a dealumination step in which silanol nests are created during acid treatment. The signal located at 2298 cm^–1^ is also conventionally attributed to strong BAS associated to the charge compensating acid protons in intraframework Al sites (Si-O(H)-Al). The successive ion exchange cycles eliminate the strong BAS, as evident from the removal of this signal in the spectra recorded for [K]Sn-USY materials. These results agree with those obtained from ammonia TPD and FTIR analyses with pyridine as the molecular probe, supporting the previous conclusions on the passivation of strong BAS with potassium after the first exchange cycle. Signals detected at 2309 and 2316 cm^–1^ are usually ascribed to the adsorption of deuterated acetonitrile onto Lewis acid sites such as those provided by tin sites incorporated to the zeolite framework. The existence of two different signals is conventionally ascribed to closed ((SiO)_4_Sn) and open ((SiO)_3_Sn-OH)^47^ configurations, respectively. Ion exchange with potassium reduces, together with BAS, the Lewis acidity too, as evidenced by the strong decrease observed in the area below the deconvolution curves attributed to tin sites. Interestingly, we do not observe any new band at 2280 cm^–1^ after ion exchange, as reported by Davis et al.^48^ on the Na exchange on Sn-β zeolites. Instead, there is a great enhancement of the intensity of the signal at 2272 cm^–1^, which could be ascribed to the overlapping of a new contribution, attributed to CD_3_CN molecules adsorbed onto very weak Lewis acid sites. This might be caused by the weakening of tin sites or by the blocking of the same with potassium sites, showing a much weaker Lewis acid strength. These results points to the interaction of potassium cations with the Sn centers, probably because of the passivation of the already commented Brønsted acid sites created by the interaction of Sn and water molecules.

Bearing in mind the observed reduction of the Lewis acid strength through pyridine and deuterated acetonitrile adsorption and FTIR analysis, a decreasing activity in the transformation of glucose into methyl lactate is expected when using Sn-USY materials prepared after several ion exchanges. Ion exchange with potassium chloride for several cycles leads, in all the cases, to active [Kxn]Sn-USY materials, yielding methyl lactate as the main product. However, strong differences can be found between the samples prepared with different ion exchange cycles, in terms of both catalytic performance and product distributions, in both cases from the beginning of the reaction. The composition of the reaction media observed at time zero—the time required to warm up the media to the reaction temperature, which takes 6 min in our experimental setup—largely varies from one catalyst to other (Figure S7). Whereas the Sn-USY parent material provides 95% substrate conversion after the warm-up step, yielding methyl glucoside as the main product, the sample prepared with a single ion exchange cycle with potassium chloride provides a different product distribution -the substrate conversion is much lower (82%) and the yields of fructose and mannose are higher as compared to the Sn-USY material-, showing a high selectivity for retro-aldol derived products. In addition, the methyl glucoside yield achieved with the single K-exchanged zeolite drops to less than 20% of the starting glucose while providing methyl lactate yields above 10%. The conversion of the substrate at time zero becomes lower with each ion exchange cycle, while the extension of the isomerization of glucose to fructose diminishes and the epimerization to mannose slightly increases. These results are consistent with those of Meier et al.,^34^ who reported an enhancement of the epimerization activity of Sn-β when ion exchanged with K_2_CO_3_. The methyl glycosidation extension at time zero becomes less and less important with each K-exchange cycle, reaching the minimum after four ion exchanges, probably because of the removal of the Brønsted acidity in this material, as previously stated. In contrast, the production of methyl lactate during the early stages of the reaction finds the maximum (13.4%) for sample [Kx2]Sn-USY being much lower for samples prepared with a higher number of ion exchange cycles. This phenomenon seems to be related to a saturation effect of the tin catalytic sites with K^+^ ions, blocking the access of the monosaccharide substrates to Sn sites and thus reducing the catalytic efficiency of the materials in retro-aldol condensation transformations.^35^

**Figure 4:**
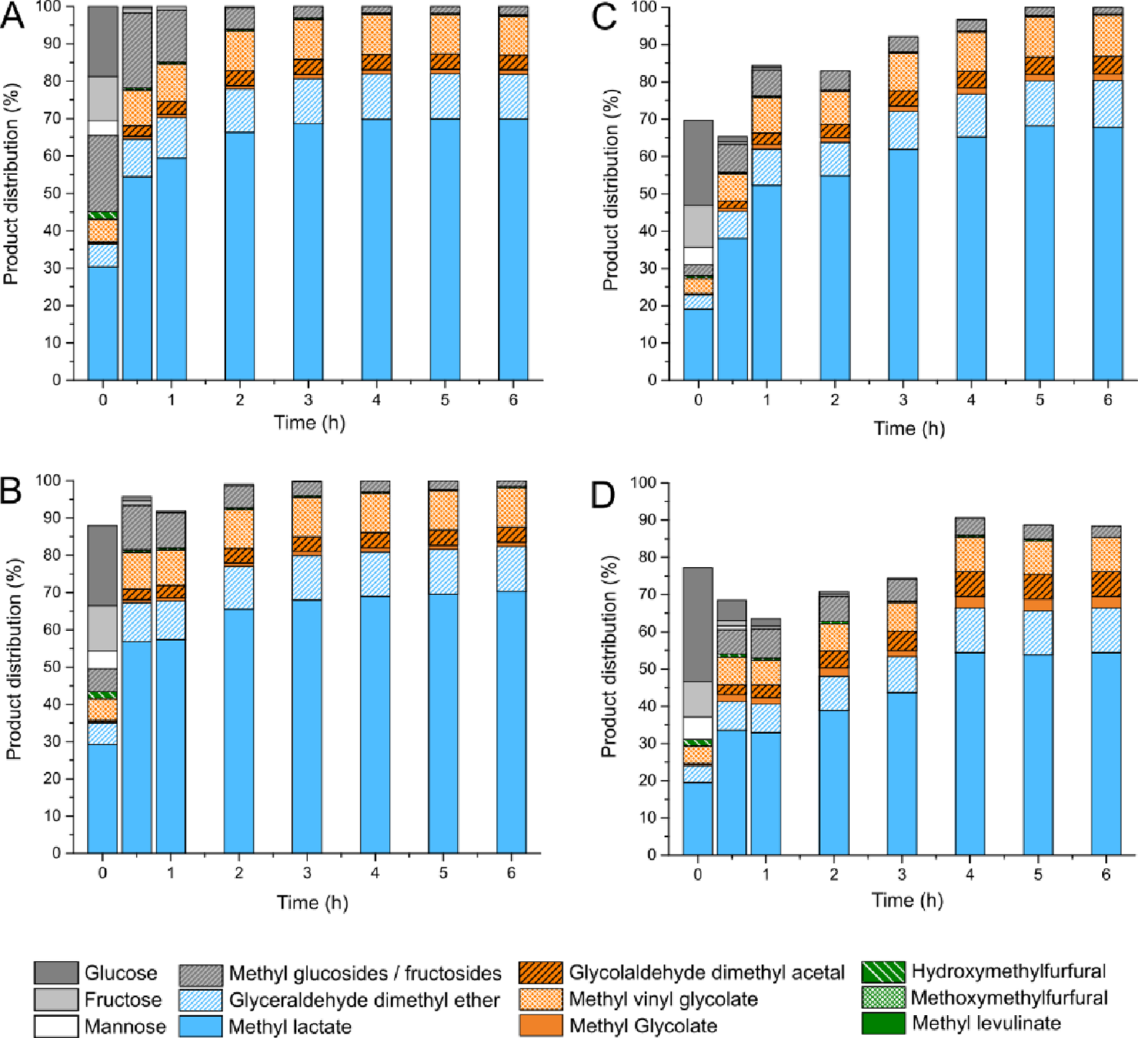
Results obtained from catalytic tests performed on methanolic solutions of glucose in the presence of K-exchanged Sn-USY zeolites: (A) [K]Sn-USY, (B) [Kx2]Sn-USY, (C) [Kx3]Sn-USY, and (D) [Kx4]Sn-USY. Reaction conditions: reaction volume, 75 mL; catalyst loading, 0.75 g; substrate concentration, 266 mM; and reaction temperature, 150 °C.

The product distributions achieved during the early steps of the catalytic assays evolve toward the rapid consumption of the different sugars (glucose, fructose, and mannose) and methyl glycosides (Figure 4). However, the consumption rate of these compounds slows down as a function of the number of ion exchange cycles. On the other side, a rapid formation of products derived from sugar retro-aldol condensation, including methyl vinyl glycolate (MVG), glycolaldehyde dimethyl acetal (GADMA), methyl glycolate (MG), and methyl lactate, is obtained (Scheme S2). From these products, C4 and C2 carbon backbone products (MVG, GADMA, and MG) are derived from the retro-aldol condensation of glucose and are present in a lower proportion as compared to the C3 methyl lactate, which is derived from the retro-condensation of fructose, being the major product in all the catalytic tests. Retro-aldol condensation products are formed in lower yields when high potassium-loaded catalysts are used, evidencing that the interaction of the alkali cations with the catalytic tin sites reduces their ability to conduct glycolytic reaction pathways. Regardless of the number of ion exchange cycles, retro-condensation products are formed at a fast rate during the first hour, while there is some availability of free sugars (glucose, fructose, or mannose). Afterward, their production rate decreases but does not stop, being formed at a substantially lower rate. Tosi et al.^41^ reported similar results for Sn-β zeolites, assigning a role of substrate masking agents to methyl glycosides, which can undergo a hydrolysis step to produce glucose/fructose, allowing the system to proceed toward retro-aldol products. This secondary route would be slower than the direct transformation of glucose into methyl lactate, and thus, it has been proposed as the origin of the production of retro-condensation products after complete substrate consumption. Nevertheless, together with the already described retro-aldol products, glyceraldehyde dimethyl ether (GDME) is also produced, being ascribed to the transformation of methyl glycosides through a retro-aldol condensation pathway followed by etherification with methanol (Scheme S2). This reaction pathway proceeds faster in the presence of the [K]Sn-USY sample and becomes less effective with the increase number of potassium exchange cycles, like the rest of the retro-aldol transformations. Unfortunately, GDME does not evolve toward the formation of methyl lactate under the tested conditions and remains stable in the reaction media, representing a dead end in the valorization of glucose.

Finally, it must be stressed that there is another difference between the K-exchanged Sn-USY zeolites in terms of catalytic performance, which is the formation of unknown products. Mass balance calculation reflects a higher mass deficit between the starting glucose and the sum of products when increasing the number of ion exchanges in the preparation of the catalyst. This deficit mainly occurs during the early stages of the catalytic tests, so side reactions leading to unknown products are most likely to be related to the transformation of the starting glucose, any of its isomers (fructose and glucose), or methyl glycosidation products. Further insights into the evolution of these products at short contact times would have to be tackled to evaluate these substrate consuming side reactions.

### Catalyst Reusability

**Figure 5:**
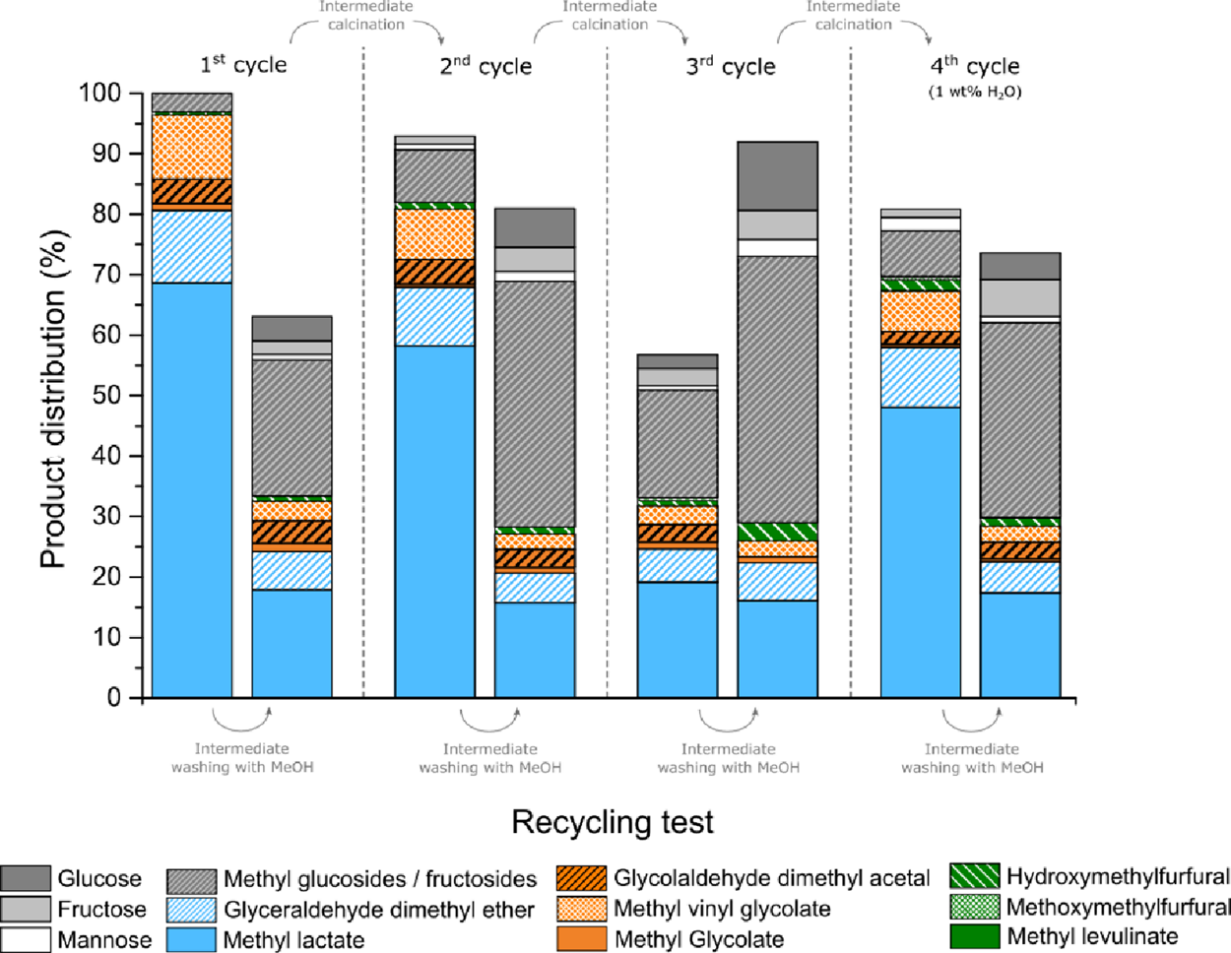
Product distribution achieved in the reutilization tests performed for sample [K]Sn-USY in the conversion of glucose in methanol at 150 °C. Reaction conditions: reaction volume, 75 mL; catalyst loading, 0.75 g; substrate concentration, 266 mM; reaction temperature, 150 °C; and reaction time, 3 h.

From the previous results, it is evident that the [K]Sn-USY material combines the required surface sites to maximize the production of methyl lactate from glucose under the tested reaction conditions. Reutilization tests have been thus performed using this sample. Figure 5 depicts the product distributions achieved in the different catalytic tests carried out using the very same catalyst sample. These tests consisted of several reaction cycles comprising the use of the catalysts in the reaction test followed by a direct reutilization after recovering the catalyst by filtration and methanol washing, both reactions performed for 3 h. The first cycle, comprising the first use and reuse, evidences the loss of the initial catalytic performance of the fresh sample. The fresh catalyst mainly led to retro-aldol condensation derived products, whereas the used catalyst provided hexoses, methyl glycosides, and a large fraction of unknown products evidenced by the significant loss of carbon balance (40% of the starting glucose). These results evidence the existence of multiple and strong deactivation phenomena taking place on the catalysts, with the formation of organic deposits, together with the zeolites’ pore blockage and consequent hindered access of the substrate to the catalytic sites, being the most plausible deactivation causes. Indeed, the surface area recorded for the spent [K]Sn-USY catalyst was 200 m^2^/g lower as compared to the fresh material. To evaluate the origin of the observed catalyst deactivation, two more reusing cycles, comprising one use and one reuse test, were carried out consecutively. Intermediate calcination in air at 550 °C was applied to regenerate the spent catalysts in between consecutive cycles, aiming for the complete removal of the organic deposits. It is important to notice that the textural properties of the spent catalyst were fully recovered after this thermal treatment. Calcination treatment seems to correct the catalyst behavior and enhance the extension of retro-condensations, although the catalytic activity recovery is not complete, and the formation of methyl glycosides increases after the calcination Reuse tests after solvent washing provide a very low production of retro-aldol condensation products but increasing quantities of sugar hexoses and methyl glycosides. This behavior is repeated in a third cycle, although the differences between the product distributions for the fresh and the calcined sample and between consecutive runs are magnified. These results suggest the existence of some other causes for catalyst deactivation apart from the occlusion of the pores by organic deposits. In this sense, Botti et al.^49^ and Padovan et al.^50^ tentatively proposed the evolution of tin sites from an open conformation ((SiO)_3_Sn-OH) toward a closed configuration ((SiO)_4_Sn) as a cause for the deactivation of Sn-β catalysts. The formation of large quantities of methyl glycosides, similarly to that occurring in the presence of the parent Sn-USY material prior to the ion exchange (Figure 2), suggests the closing of the tin catalytic sites. However, several authors have suggested that the closing of tin sites could be avoided by the addition of water to the reaction media.^49,50^ In this way, we have attempted a fourth reuse cycle by using the catalysts in the presence of slightly modified reaction media, including 1 wt % of water referred to methanol. Under these conditions, the catalysts demonstrated an enhanced catalytic activity in the promotion of retro-aldol condensations as compared to the previous runs, although it was not comparable to that shown by the fresh catalyst. Nevertheless, reuse tests evidenced the existence of other causes of deactivation that are not eliminated by the addition of water. For instance, the ICP-OES analysis of the spent catalyst after the eight consecutive reaction tests evidenced the loss of 80% of the starting potassium; thus, the promotion effect of these cations on the catalytic activity of tin sites in retro-aldol condensation is also lost. Also, the formation of carbonaceous deposits onto the catalyst is still taking place even in the modified reaction media. Nevertheless, our results evidence that some of the deactivation causes that limit the reusability of these catalysts can be partially overcome by the control of the reaction media to preserve the integrity of the catalyst sites.

## Conclusions

Tin-containing USY zeolites were prepared through a highly efficient post-synthetic metalation process comprising a soft dealumination and the grafting of isolated tin species, reaching 2 wt % of the metal without the formation of tin oxide. The modification of the Sn-USY zeolite by ion exchange with alkali chlorides led to the passivation of strong Brønsted acid sites, modifying the catalytic performance of Sn-USY zeolites in the transformation of glucose to alkyl lactates. The potassium-exchanged Sn-USY zeolite demonstrated an outstanding catalytic performance and selectivity, providing up to 70% of the starting glucose as the target hydroxyester. Moreover, retro-aldol condensation derived products totalized an overall product yield above 95% of the starting sugar. This unprecedented result makes the Sn-USY material a promising alternative to Sn-β, a much more expensive zeolite structure, in the transformation of sugar monosaccharides to alkyl hydroxyesters. Attempts to enhance the beneficial influence of K-exchange in the catalytic efficiency of the tin-containing USY zeolites by increasing K loadings led to the reduction of the Lewis acidity of the material and their catalytic performance in the treatment of methanolic solutions of glucose. Finally, reutilization tests performed with K-exchanged Sn-USY evidenced the existence of multiple deactivation phenomena, which could be partially alleviated through different alternatives, including catalyst calcination or the use of minimal amounts of water in the reaction media.

## Supporting Information Available

Experimental section details; schematic representation of the grafting process; reaction network; physicochemical properties of the samples; acid capacity from Py FTIR; ^27^Al solid-state MAS NMR analysis; XRD patterns, N_2_ adsorption isotherms, and pore size distribution; NH_3_ TPD; FTIR spectra in the OH stretching region; acid concentration calculated from Py FTIR; and initial product distributions (PDF)

## Abbreviations

FAU: faujasite
USY: ultra-stable Y zeolite
TEA: triethylamine
MLA: methyl-d-lactate
MG: methyl glycolate
GADMA: glycolaldehyde dimethyl acetal
MLE: methyl levulinate
GLU: glucose
FRU: fructose
MAN: mannose
MGP: methyl-d-glucopyranoside
BAS: Brønsted acid site
LAS: Lewis acid site
5-HMF: 5-hydroxymethyl furfural
GDME: glyceraldehyde dimethyl ether
