# Supplementary material for: Efficient Conversion of Glucose to Methyl Lactate with Sn-USY: Retro-aldol Activity Promotion by Controlled Ion Exchange
Source: ACS Sustain Chem Eng. 2022 Jun 28;10(27):8885–96. doi: 10.1021/acssuschemeng.2c01987 (PMC9278086; doi:10.1021/acssuschemeng.2c01987)
Supplement: Supplementary file 1 — sc2c01987_si_001.pdf [file sc2c01987_si_001.pdf]

SUPPLEMENTARY INFORMATION

Efficient conversion of glucose to methyl lactate with Sn-USY: catalyst activation by ion exchange

Jose M. Jimenez-Martin,[†] Ana Orozco-Saumell,[‡] Héctor Hernando,[⸰] María Linares,[†] Rafael Mariscal,[‡] Manuel López Granados,[‡] Alicia García,[†] and Jose Iglesias[†,*]

† Chemical & Environmental Engineering Group. Universidad Rey Juan Carlos. C/ Tulipan s/n, 28933. Madrid, Spain.

‡ Energy and Sustainable Chemistry (EQS) Group. Institute of Catalysis and Petrochemistry, CSIC, C/ Marie Curie 2, Campus de Cantoblanco. 28049 Madrid, Spain

⸰ IMDEA Energy Institute, A. Ramón de la Sagra 3, 28935, Móstoles, Madrid, Spain

Number of pages: 18

Number of schemes: 2

Number of tables: 2

Number of figures: 7

Table of contents

Experimental section S3

Materials S3

Catalyst preparation S3

Catalyst characterization S5

Catalytic activity tests S6

Scheme S1. Schematic representation of the experimental process S8

Scheme S2. Reaction network S9

Table S1. Physico-chemical and textural properties of the samples S10

Table S2. Acid capacity from Py FTIR S11

Figure S1. ^{27}Al solid state MAS NMR analysis S12

Figure S2. XRD patterns, N_2 adsorption isotherms and Pore size distribution S13

Figure S3. NH_3 TPD S14

Figure S4. FTIR spectra in the OH stretching region S15

Figure S5. Acid concentration calculated from Py FTIR S16

Figure S6. Initial product distributions S17

Experimental

Materials

Commercial USY zeolite (CBV 712, Zeolyst International) was used as parent material for the synthesis of Sn-USY materials. Nitric acid (HNO_3, Sigma Aldrich, 70%) was used in the dealumination process. Dichloromethane (CH_2Cl_2, Scharlab), tin(IV) chloride pentahydrate ($SnCl_4 \cdot 5H_2O$, 98%, Alfa Aesar), and triethylamine ($N(CH_2CH_3)_3$, TEA, 99%, Sigma Aldrich) were used in the metalation procedure as solvent, tin source, and grafting catalyst, respectively. Lithium, sodium and potassium chloride (99%, Sigma Aldrich) were used as alkali cation sources in ion exchange treatments. D-(+)-Glucose (GLU, 99%, Sigma Aldrich), and methanol (CH_3OH, HPLC Grade, Sharlab) were used in catalytic tests as reaction substrate and solvent, respectively. Methyl D-lactate (MLA, 99%, Sigma Aldrich), methyl glycolate (MG, 98%, Sigma Aldrich), methyl vinylglycolate (MVG, 98.4%, Apollo Scientific), glycolaldehyde dimethyl acetal (GADMA, 98%, Alfa Aesar), methyl levulinate (MLE, 98%, Sigma Aldrich), methoxymethylfurfural (MMF, 95%, abcr GmbH) and hydroxymethylfurfural (HMF, 98%, abcr GmbH) were used as standards for the calibration of a gas chromatography unit, using decane ($C_{10}H_{22}$, 98%, Honeywell) as an internal standard. D-(+)-glucose (GLU, 99%, Sigma Aldrich), D-(-)-fructose (FRU, 99%, Sigma Aldrich), D-(+)-mannose (MAN, 99%, Sigma Aldrich) and methyl-D-glucopyranoside (MGP, 99%, Sigma Aldrich) were all used as standards for the calibration of a HPLC unit.

Catalyst preparation

Sn-USY materials were prepared via post-synthetic catalytic metalation of dealuminated USY with tin species. Commercial USY zeolite was dealuminated by contacting the parent material with aqueous HNO_3 solution (10 M, 20 $mL \cdot g^{-1}$ of zeolite) for 1 hour at room temperature. The treated solids were recovered by centrifugation, thoroughly washed with deionized water until neutral pH, and dried at 110ºC overnight. This procedure was repeated twice to ensure the removal of a large fraction of the starting aluminium loading.

The metalation process consisted of incorporating tin species to the dealuminated zeolite by reacting the hydroxyl functionalities created in aluminium vacancies, with tin (IV) chloride. For this purpose, the dealuminated zeolite was previously dried by refluxing the material in dichloromethane (40 mL·g^{-1} of zeolite), using a Dean and Stark apparatus for solvents heavier than water, operated under N_2 atmosphere. This procedure allowed removing the water molecules adsorbed onto the surface of the dealuminated zeolite at very low temperature (ca 38°C) through an azeotropic distillation procedure, thus preserving the integrity of a high population of hydroxyl groups. Once dried, the dealuminated zeolite was contacted with $SnCl_4$ (0.06 g·g^{-1} of zeolite), and the resultant suspension was stirred for 5 hours at room temperature in N_2 atmosphere. This prolonged contact time has the purpose to allow the diffusion of the tin source inside the porous structure of the zeolite and to intimate contact the metal precursor with the zeolite support. Afterwards, trimethylamine (TEA) was added (TEA:$SnCl_4$ = 4 molar ratio) to chemically assist the Sn incorporation, in a similar way to that previously reported by Maschmeyer *et al.* for titanocene dichloride.[42] In this step, triethylamine acts as an activator of the metal grafting by activating the silanol groups at the dealuminated zeolite, which are readily attacked by the tin species, creating Sn-O-Si bonds. As a result, hydrogen chloride evolving from the reaction is neutralized by TEA to create the adduct triethylamine hydrochloride (See scheme SI-1), which is soluble in CH_2Cl_2. The zeolite was then recovered by centrifugation and dried at room temperature before subjecting the material to air calcination (200 °C, 1.8 °C·min^{-1}, 6 h and 550 °C, 1.8 °C·min^{-1}, 6 h).

The calcined material was modified by successive ion exchange, obtaining the denoted [Mxn]Sn-USY (where M indicates the cation, and n the number of ion exchange cycles if higher than 1). The ion exchange process consisted of suspending the Sn-USY material in an aqueous solution of LiCl, NaCl or KCl (0.5 M, 100 mL·g^{-1} of zeolite) for 2 hours at 50 °C. This contact time was selected to ensure the complete passivation of the existing Brønsted acid sites, based on the evolution of the pH of the ion exchange media, which reached a stable value after contacting the Sn-USY zeolites with the alkali chloride solutions. The samples were then recovered by centrifugation, and thoroughly

washed (twice) with deionized water (stirring for 10 minutes at 50ºC) to remove the excess of alkaline metal chloride. In this way, only metal ions strongly interacting with the Sn-zeolites remain attached to the material. Finally, samples were dried overnight at 110 °C and air calcined at 550 °C for 6 h in static air.

Catalyst characterization

Structural properties of the zeolite samples were evaluated by X-ray powder diffraction (XRD). XRD patterns were recorded with a Panalytical X'Pert Pro unit using the Cu Kα line in the 2θ angle range of 5-90°. Metal contents (Al, Sn, and alkaline metal cations) were determined by ICP-OES using a Varian 720-ES spectrophotometer, upon previous calibration with standard stock solutions. Nitrogen adsorption-desorption isotherms were recorded at 77 K using a Micromeritics Tristar 3000 system and argon adsorption-desorption isotherms were measured at 87 K with a Micromeritics Asap 2020 instrument. The surface area was determined applying the BET equation. The t-plot method was used to determine the micropore volume of the samples. The pore size distributions were calculated from the adsorption branch of the argon isotherms using the NLDFT model and a Kernel function with cylindrical pore geometry (Quantachrome). Ammonia and CO_2 temperature programmed desorption measurements were carried out using a Micromeritics AUTOCHEM II 2920. Prior to the analysis, the samples were outgassed in He flow (50 NmL·min^{-1}) by heating at a rate of 10 °C·min^{-1} up to 550 °C, and keeping the sample at this temperature for 30 min. After cooling down to a certain temperature (180ºC for NH_3; 50ºC for CO_2), a stream of the molecular probe in helium (NH_3 - 0.5%; CO_2 – 5%) of 50 NmL·min^{-1} was passed through the sample for 30 min to ensure saturation of acid or basic sites. The physisorbed molecular probes were removed by flowing He at the adsorption temperature for 60 min. The chemically adsorbed ammonia or CO_2 were determined by increasing the temperature up to 550ºC with a heating rate of 10ºC·min^{-1}, this temperature being maintained constant for 30 min. A thermal conductivity detector was used to record the evolution of ammonia or CO_2 in the effluent stream. Diffuse reflectance UV-Vis spectra were carried out with a double beam Varian Cary 5000 spectrophotometer in the spectral range of 200-600 nm. Thermal treatments were conducted in a high-

temperature catalytic reaction chamber (HVC-DRP Harrick Scientific Products, NY). The spectra of the as-prepared fresh samples were obtained directly, whereas the thermal treatment analyses were performed by heating the samples to 350 °C (ramp: 5 °C·min^{-1}) for 1 h in air. The DR UV-Vis spectra of the treated samples were recorded at 350 °C to prevent rehydration. Deuterated acetonitrile (CD_3CN) and pyridine (Py) were used as molecular probes to assess the acidity of the samples. CD_3CN diffuse reflectance infrared Fourier transform (DRIFT) spectra were collected in a Nicolet 5700 spectrometer fitted with a Hg–Cd–Te cryodetector, using a catalytic chamber fitted with a temperature controller that allowed in situ thermal treatments (Harrick Scientific Products) using a praying mantis as a mirror optical accessory. Prior to CD_3CN DRIFT spectra collection, samples were treated at 120 °C for 1 h under argon flow (50 mL·min^{-1}) as a surface cleaning step. Argon saturated in CD_3CN was passed through the sample until saturation at room temperature. The fraction of physically CD_3CN was removed by flushing the chamber with Ar flow. DRIFT spectra were recorded in the spectral range of 2400–2200 cm^{-1} with a resolution of 4 cm^{-1}. Py FTIR spectra were collected using the buffer technique (13 mm, 8-15 mg/cm^2; dried at 70ºC overnight and activated at 500ºC for 2 h prior to their use) in a Jasco FTIR-4600 unit fitted with a TGS detector and a transmission cell with CaF_2 windows. Samples were treated with Py at 150ºC and 4 mbar to adsorb the molecular probe prior their analysis. Spectra were collected in the range 4000-1000 cm^{-1} with a resolution of 4 cm^{-1}.

Catalytic activity tests

The catalytic performance of the Sn-USY materials was evaluated in the direct transformation of glucose into methyl lactate. The catalytic tests were carried out in a 100 mL stainless steel reactor (Autoclave Engineers/Parker Hannifin) fitted with a temperature control system, mechanical stirring, and a pressure transducer. Typically, 3.6 g of glucose, 75.5 mL of methanol, 0.75 g of catalyst, and decane (internal standard, 0.01 g·mL^{-1}), were placed together in the reactor vessel. After sealing the reactor and fluxing the reaction atmosphere with N_2, the temperature (150°C) and the stirring rate conditions (500 rpm) were set, considering time zero once the temperature set point was reached. Sample aliquots were periodically withdrawn from the reaction media over 6 h, using a water-cooled

samples collecting system. Recycling catalytic tests were performed using spent catalysts instead of fresh samples, which were recovered by filtration from previous catalytic assays, washed with fresh methanol at room temperature, or calcined in air at 550ºC, prior to its reuse.

Reaction samples were analyzed by means of GC and HPLC using a Varian CP3900 GC unit, fitted with a CP-WAX-52CB column, and an Agilent 1260 unit, fitted with Diode Array and Light Scattering detectors and a Shodex NH2P-50-4E column. Product quantification was performed after previous calibration of the chromatography units with standard stock solutions. GC-MS were used to identify unknown products using a Bruker 320-MS GC Quadrupole Mass Spectrometer fitted BR-SWax column (30 m x 0.25 mm, DF=0.25 μm). Substrate conversion (X) and product yields (Yi) were calculated as follows:

$$X(\%) = \frac{Reacted\ moles\ of\ glucose}{Initial\ moles\ of\ glucose} \cdot 100 \qquad (1)$$

$$Y_i(\%) = \frac{Carbon\ atoms\ in\ product\ i \cdot Produced\ moles\ of\ product\ i}{Carbon\ atoms\ in\ glucose \cdot\ Initial\ moles\ of\ glucose} \cdot 100 \qquad (2)$$

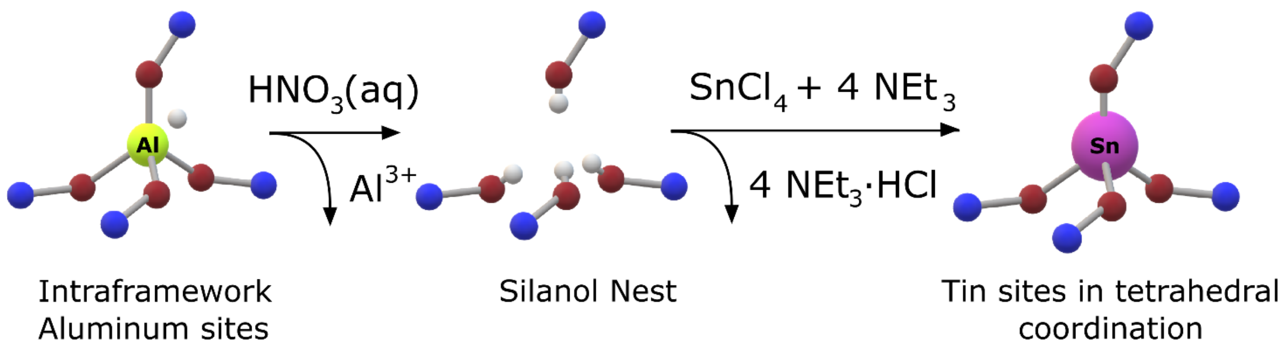

Scheme S1. Schematic representation of the acid dealumination and metalation of USY zeolites with tin species.

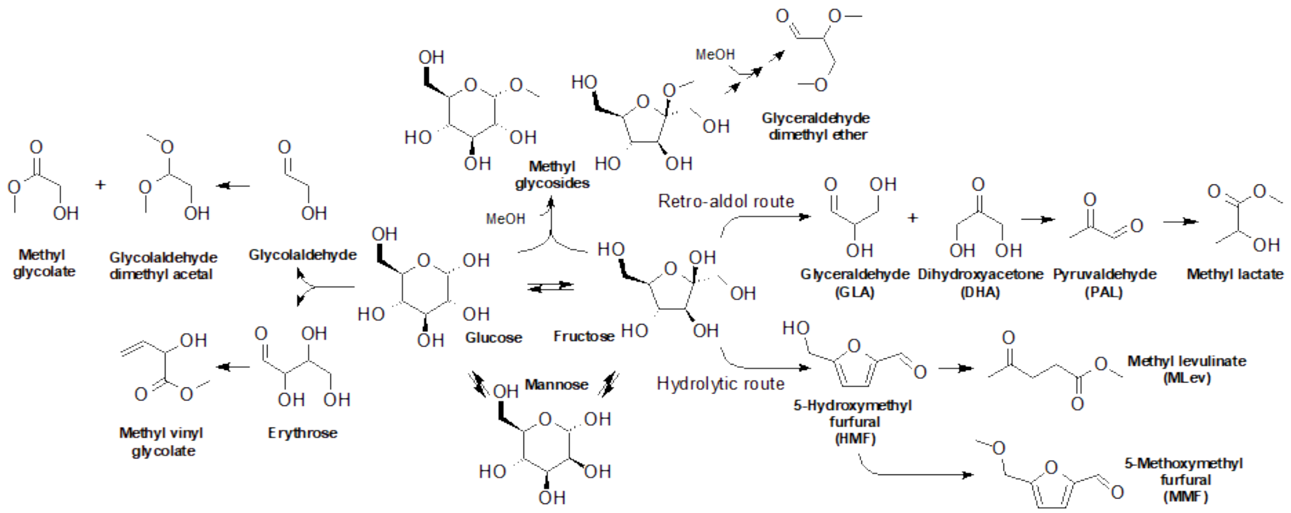

Scheme S2. Reaction network taking place when treating methanolic solutions of glucose in the presence of Sn-USY zeolites.

Table S1. Physico-chemical properties of the of Sn-USY zeolites prepared in this work

Sample	Al [a] (wt %)	Sn [a] (wt %)	M [a, b] (wt %)	M : Sn [c] (at : at)	Acidity [d] ($meqH^{+}\cdot g^{-1}$)	T_{max} [e] (°C)	S_{BET} [f] ($m^2\cdot g^{-1}$)	S_{μ} [g] ($m^2\cdot g^{-1}$)	S_{EXT} [h] ($m^2\cdot g^{-1}$)	V_t [i] ($cm^3\cdot g^{-1}$)
**H-USY**	5.47	0.00	0.00	0.00	3.4	318	758	601	157	0.441
**Sn-USY**	0.58	2.09	0.00	0.00	2.8	280	786	570	216	0.499
**[Li]Sn-USY**	0.43	2.01	0.03	0.25	2.4	256	799	570	229	0.487
**[Na]Sn-USY**	0.41	2.00	0.17	0.43	2.1	261	812	568	243	0.504
**[K]Sn-USY**	0.55	1.91	0.40	0.64	1.8	257	830	564	265	0.499
**[Kx2]Sn-USY**	0.57	2.00	0.61	0.93	1.4	257	835	486	349	0.509
**[Kx3]Sn-USY**	0.56	2.11	0.76	1.09	1.2	270	829	497	331	0.525
**[Kx4]Sn-USY**	0.59	2.09	0.85	1.24	1.1	252	822	467	355	0.534

[a] Metal loading measured by ICP-OES; [b] M = alkali metal; [c] Alkali to Sn atom ratio; [d] Acid capacity calculated from NH_3-TPD experiments; [e] Desorption temperature recorded for the maximum in ammonia-TPD curves; [f] Specific surface area determined by the BET method using the Rouquerol criteria for zeolites; [g] micropore surface area determined by the t-plot method; [h] external surface area calculated as $S_{EXT} = S_{BET} - S_m$; [i] Total pore volume recorded at $P/P_0 = 0.9$

Table S2. Acid capacity calculated for Sn-USY materials by means of FTIR using pyridine as molecular probe.

Sample	Temperature	150ºC	250ºC	350ºC	450ºC
**Sn-USY**	B ($mmol \cdot g^{-1}$)	0.063	0.059	0.051	0.032
	L ($mmol \cdot g^{-1}$)	0.230	0.067	0.023	0.005
**[K]Sn-USY**	B ($mmol \cdot g^{-1}$)	0.025	0.023	0.012	0.010
	L ($mmol \cdot g^{-1}$)	0.119	0.031	0.006	0.003
**[Kx2]Sn-USY**	B ($mmol \cdot g^{-1}$)	0.016	0.016	0.014	0.013
	L ($mmol \cdot g^{-1}$)	0.129	0.029	0.005	0.001
**[Kx3]Sn-USY**	B ($mmol \cdot g^{-1}$)	0.015	0.015	0.015	0.011
	L ($mmol \cdot g^{-1}$)	0.127	0.025	0.004	0.005
**[Kx4]Sn-USY**	B ($mmol \cdot g^{-1}$)	0.063	0.059	0.051	0.032
	L ($mmol \cdot g^{-1}$)	0.230	0.067	0.023	0.005

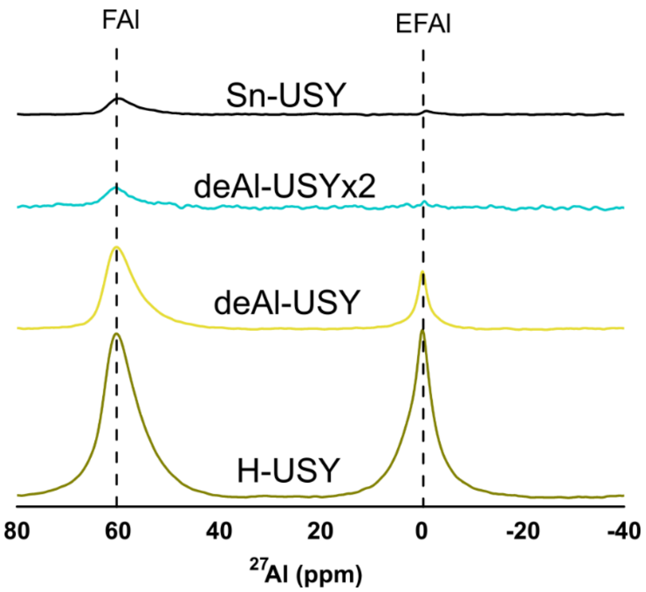

Figure S1. ^{27}Al solid state MAS NMR analysis recorded for parent H-USY and the materials obtained thereof by dealumination and metalation with tin (2wt%)

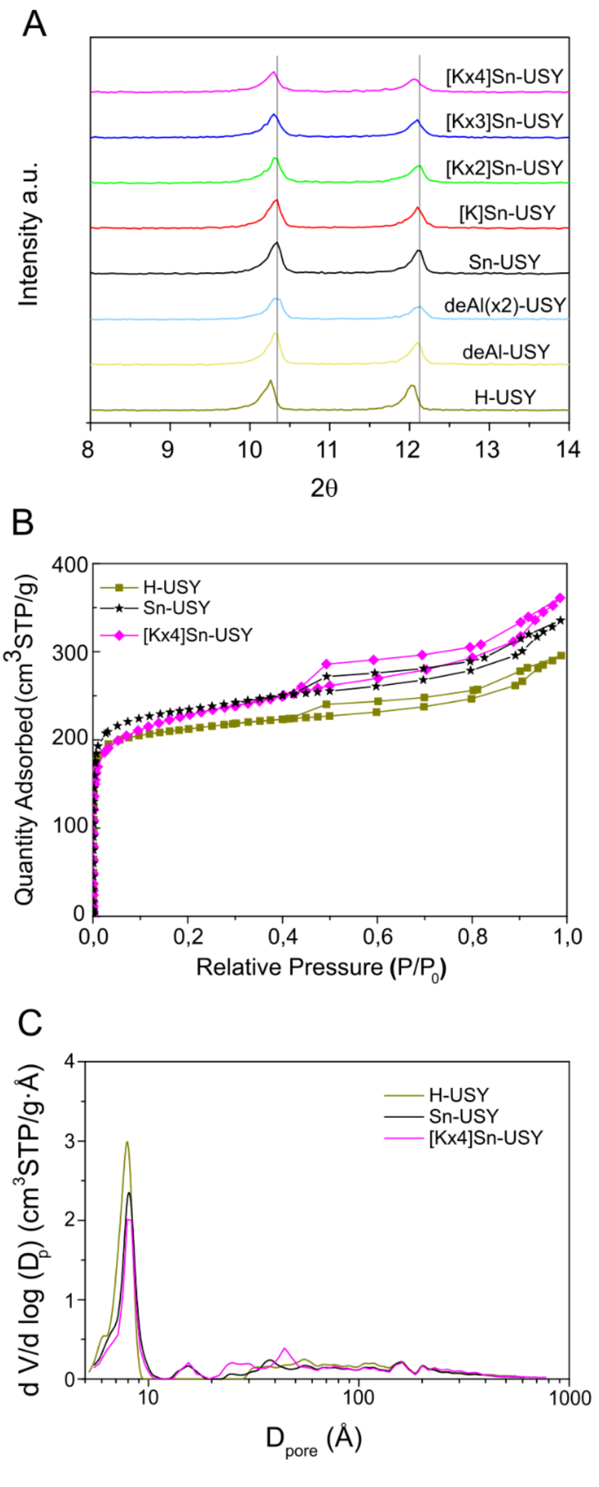

Figure S2. A) XRD patterns, B) N_2 adsorption isotherms and C) Pore size distribution for the prepared Sn-USY materials

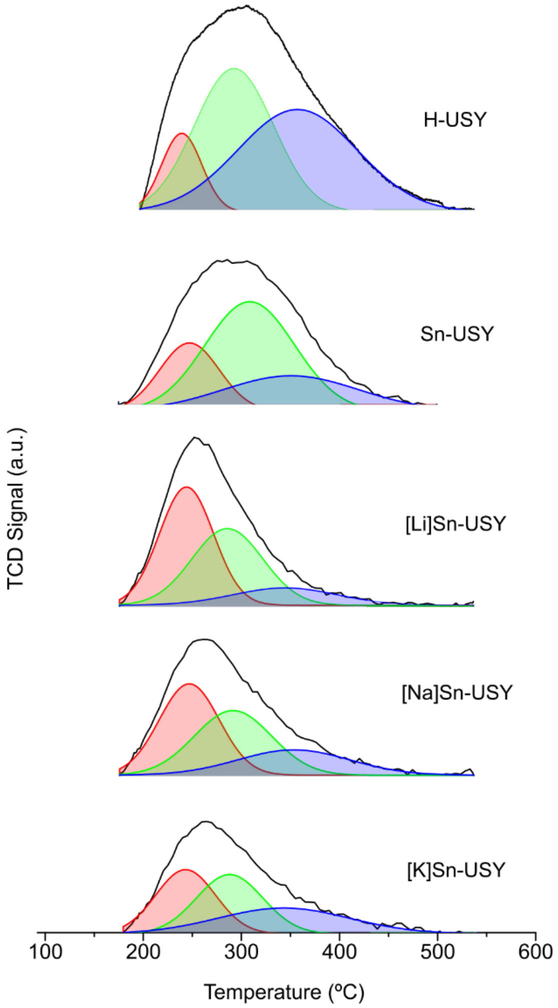

Figure S3. NH_3 TPD recorded for the starting USY, parent Sn-USY and Li, Na and K ion exchanged Sn-USY zeolites

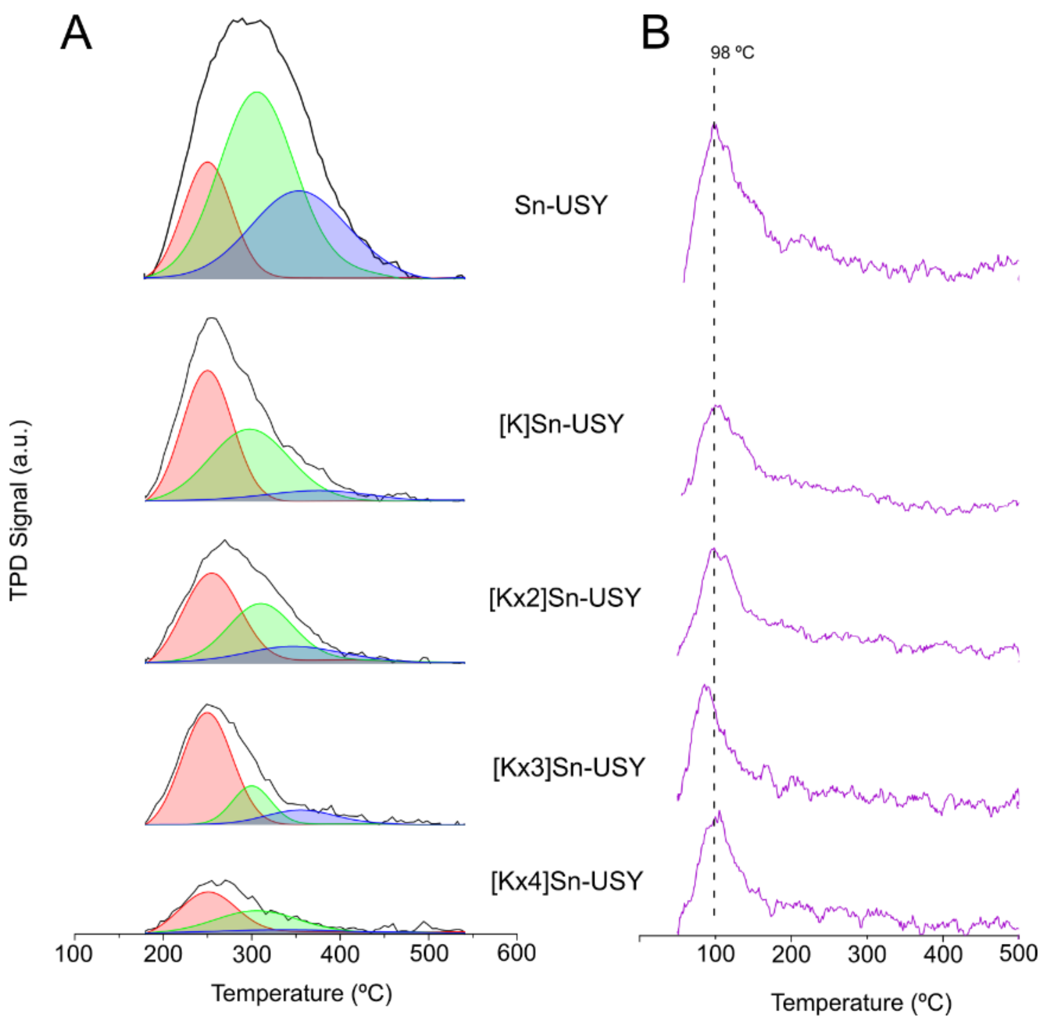

Figure S4. Thermal Programmed Desorption Experiments conducted to evaluate total acidity and basicity capacities in Sn-USY materials and K-exchanged samples. A) NH_3 TPD and B) CO_2 TPD.

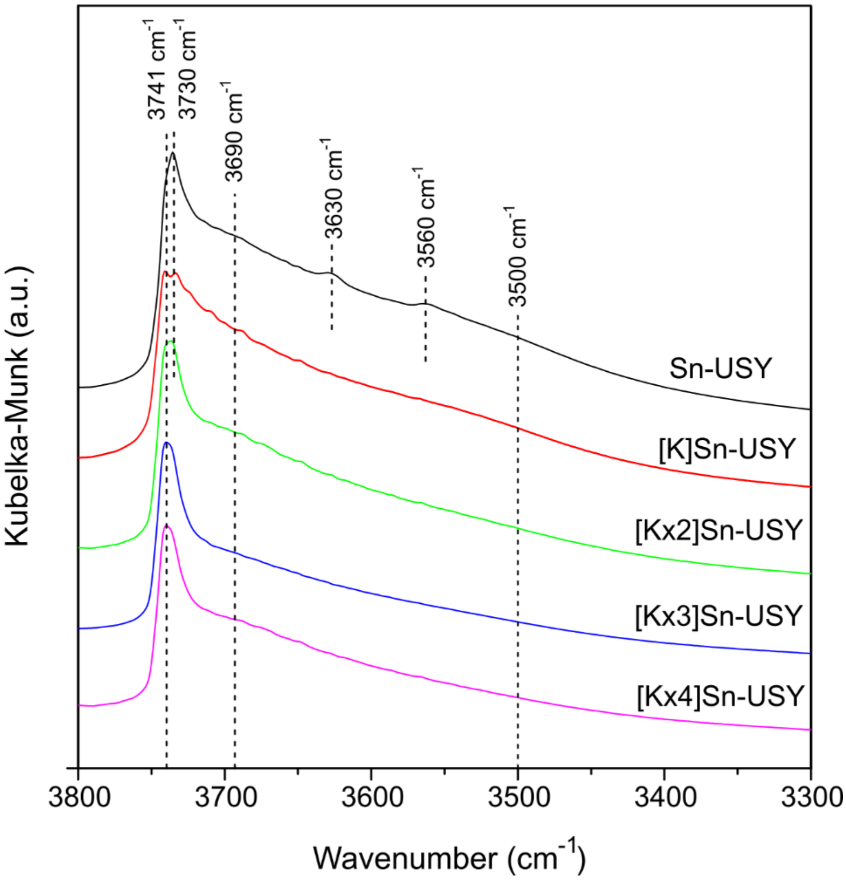

Figure S5. Infrared spectra in the OH stretching region recorded for Sn-USY zeolite and samples prepared by K-exchange thereof.

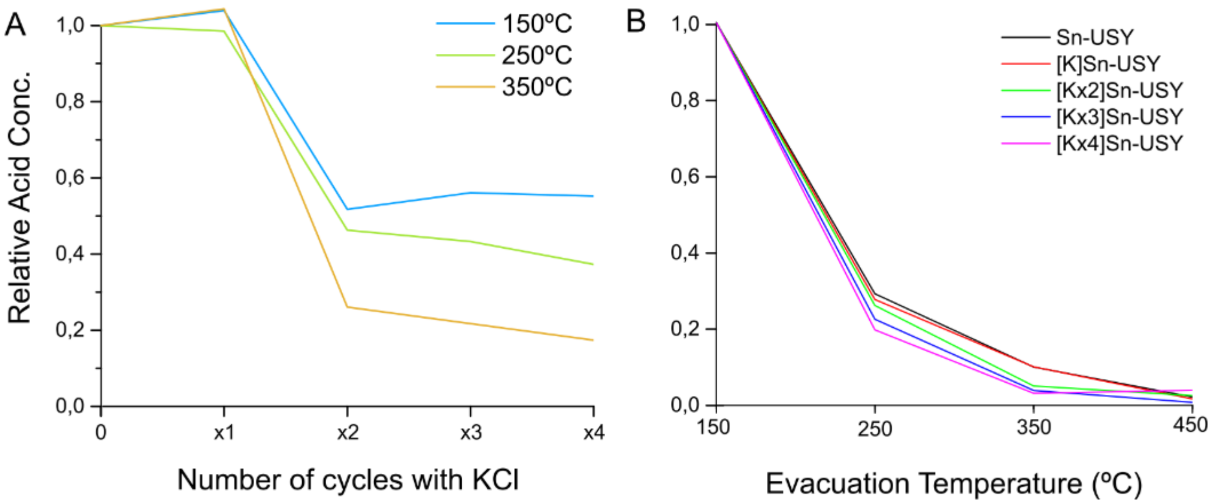

Figure S6. Relative acid concentration calculated from Py FTIR for ion exchange Sn-USY samples A) as a function of ion exchange cycles and B) as a function of evacuation temperature.

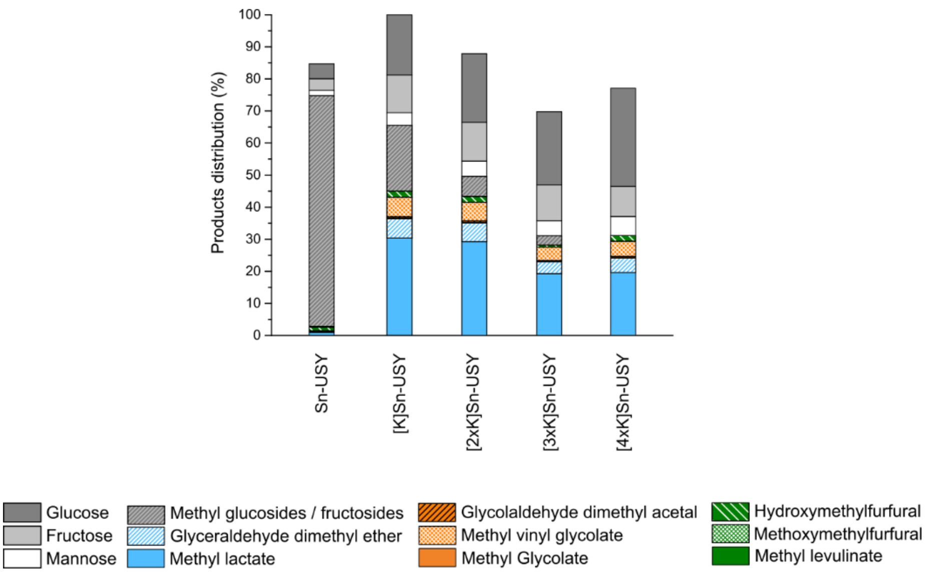

Figure S7. Initial product distributions achieved at time zero for Sn-USY materials in the treatment of methanolic solutions of glucose. Reaction conditions: reaction volume 75 mL; catalyst loading 0.75 g; substrate concentration 360 mM; reaction temperature 150ºC
